# Proteomic analysis of protein lysine 2-hydroxyisobutyrylation (K_hib_) in soybean leaves

**DOI:** 10.1186/s12870-022-04033-6

**Published:** 2023-01-12

**Authors:** Wei Zhao, Ting-Hu Ren, Yan-Zheng Zhou, Sheng-Bo Liu, Xin-Yang Huang, Tang-Yuan Ning, Geng Li

**Affiliations:** 1 College of Agronomy, Shandong Agricultural University, Tai’an, Shandong 271018 People’s Republic of China; 2 Jining Academy of Agricultural Sciences, Jining, Shandong 272075 People’s Republic of China

**Keywords:** Post-translational modification (PTM), Lysine 2-hydroxyisobutyrylation (K_hib_), Soybean, Proteomics, Motif

## Abstract

**Background:**

Protein lysine 2-hydroxyisobutyrylation (K_hib_) is a novel post-translational modification (PTM) discovered in cells or tissues of animals, microorganisms and plants in recent years. Proteome-wide identification of K_hib_-modified proteins has been performed in several plant species, suggesting that K_hib_-modified proteins are involved in a variety of biological processes and metabolic pathways. However, the protein K_hib_ modification in soybean, a globally important legume crop that provides the rich source of plant protein and oil, remains unclear.

**Results:**

In this study, the K_hib_-modified proteins in soybean leaves were identified for the first time using affinity enrichment and high-resolution mass spectrometry-based proteomic techniques, and a systematic bioinformatics analysis of these K_hib_-modified proteins was performed. Our results showed that a total of 4251 K_hib_ sites in 1532 proteins were identified as overlapping in three replicates (the raw mass spectrometry data are available via ProteomeXchange with the identifier of PXD03650). These K_hib_-modified proteins are involved in a wide range of cellular processes, particularly enriched in biosynthesis, central carbon metabolism and photosynthesis, and are widely distributed in subcellular locations, mainly in chloroplasts, cytoplasm and nucleus. In addition, a total of 12 sequence motifs were extracted from all identified K_hib_ peptides, and a basic amino acid residue (K), an acidic amino acid residue (E) and three aliphatic amino acid residues with small side chains (G/A/V) were found to be more preferred around the K_hib_ site. Furthermore, 16 highly-connected clusters of K_hib_ proteins were retrieved from the global PPI network, which suggest that K_hib_ modifications tend to occur in proteins associated with specific functional clusters.

**Conclusions:**

These findings suggest that K_hib_ modification is an abundant and conserved PTM in soybean and that this modification may play an important role in regulating physiological processes in soybean leaves. The K_hib_ proteomic data obtained in this study will help to further elucidate the regulatory mechanisms of K_hib_ modification in soybean in the future.

**Supplementary Information:**

The online version contains supplementary material available at 10.1186/s12870-022-04033-6.

## Background

Protein post-translational modification (PTM) is known to be the most effective strategy for altering protein properties, such as stability, activity, cellular localization, and protein–protein interactions [1, 2], and play key roles in a variety of biological processes and metabolic pathways [3, 4]. Currently, more than 400 different types of PTMs have been discovered in various organisms [5]. Among these PTMs, lysine residue of proteins has been found to be one of the most common modification sites, where acylation modification of lysine is a type of modification that has been extensively studied in recent years, including methylation (K_me_), acetylation (K_ac_), crotonylation (K_cr_), butyrylation (K_bu_), glutarylation (K_glu_), succinylation (K_succ_), malonylation (K_mal_), propionylation (K_pr_), ubiquitination (K_ub_) and 2-hydroxyisobutyrylation (K_hib_) [6, 7]. In recent years, due to the development of high-resolution mass spectrometry-based proteomic techniques, proteome-wide identification and analysis of lysine acylation has been performed in a variety of organisms, including eukaryotes and prokaryotes, and these types of lysine acylation modifications have been found to be a family of PTMs that are physiologically relevant [2, 8].

2-Hydroxyisobutyrate is a short-chain fatty acid present in a large number of biofluids in humans, including blood, urine, and feces [2] and is a precursor for the synthesis of 2-hydroxyisobutyryl coenzyme A [8]. The lysine 2-hydroxyisobutyrylation (K_hib_) modification is a new protein PTM, which was first identified on histones from mammalian cells by Dai et al. in 2014 [9]. However, recent studies have shown that this modification also occurs in nonhistone proteins in various compartments of cells. To date, K_hib_ modification has been identified in human or animal [9–13], fungus (*Aspergillus flavus*[14], *Fusarium graminearum*[15], *Candida albicans*[16], bacteria (*Proteus mirabilis*[17] and *Toxoplasma gondii*[18]) and plants. All these studies indicate that K_hib_ modification is not only widely present in various organisms, but also involved in a wide variety of physiological functions.

In plants, the global identification of K_hib_-modified proteins has been carried out in several different species, such as rice (*Oryza sativa* L.) [19, 20], wheat (*Triticum aestivum* L.) [5, 21, 22], rhubarb (*Rheum palmatum* L.) [23], *Physcomitrella patens*[24] and *Arabidopsis siliques*[7]. In wheat, 6328 K_hib_ sites in 2186 proteins were identified in three replicates from the roots of cultivar Jimai 44 [5]. However, in the leaves of this wheat cultivar, only 3004 K_hib_ sites in 1104 proteins were identified [22]. While a similar number of K_hib_ sites and K_hib_-modified proteins were identified in the leaves of wheat cultivar Ak58, which identified 3348 K_hib_ sites in 1074 proteins [21]. In addition, in the leaves of Chinese herb rhubarb, 4333 overlapping K_hib_ sites in 1525 proteins were identified in three independent tests [23]. These results indicate that the number of K_hib_ sites and K_hib_-modified proteins in leaves is much lower than that in roots. Moreover, the K_hib_-modified proteins whether identified in wheat roots and leaves, rhubarb leaves, or rice flowers and seedlings are all distributed in various compartments of the cell, with most K_hib_-modified proteins located in the chloroplast, cytoplasm and nucleus. Meanwhile, the K_hib_-modified proteins identified in these plants show a variety of functions, such as binding, catalytic activity, and sensitivity of structural molecules [20, 23]. Based on the accumulated research evidence of K_hib_ modification, this modification is considered to be widely present in plant proteins and is an evolutionarily conserved PTM [19, 23].

Soybean [*Glycine max* (L.) Merr.] is an important legume crop that is widely grown worldwide [25]. As one of the most important economic crops in the world, soybean provides an abundant source of plant protein and oil for human and livestock, accounting for approximately 56% of global oilseed production [26]. The leaf is an important organ of the plant and is considered the energy factory of the plant, which efficiently absorbs energy from sunlight and converts it into bioenergy through photosynthesis, thus, the leaf largely determines the yield of soybean [27]. Therefore, a better understanding of the function of leaf proteins will provide the basis for research to improve soybean yields.

A previous study on the global analysis of lysine acetylation (K_ac_) modification in soybean leaves has shown that this modification is involved in the regulation of proteins related to ribosome activity, protein biosynthesis, carbohydrate and energy metabolism, photosynthesis and fatty acid metabolism [28]. However, the K_hib_ modification in soybean leaves has not been investigated. To better understand the physiological role of soybean leaves and the potential regulatory function of K_hib_ modification on its physiological activity, we performed the first systematic analysis of protein K_hib_ modification in soybean leaves. Our data suggest that K_hib_ modification is widely present in proteins of soybean leaves and is involved in various biological processes. The K_hib_ sites and proteins identified in this study not only expand our understanding of the functional role of K_hib_ modification, but also provide a platform for further exploration of K_hib_ modification in soybean physiology and biology.

## Results and discussion

### Global identification of K_hib_ modification in soybean leaves

The K_hib_ modification is a newly discovered modification first found on histone proteins in mammalian cells [9]. Since then, this protein modification has been found in various organisms, including animals, microorganisms and plants. In plants, proteome-wide K_hib_ modifications have been profiled in *Arabidopsis siliques*[7], rhubarb [23], wheat [5, 22], rice [20, 29], etc. Soybean is an important economic crop grown worldwide [25]. Previous studies have reported global analysis of protein K_ac_modifiaction in soybean seeds [30] and leaves [28] and found this modification participate in various biological processes. However, K_hib_ modification has not been identified in soybean. To gain a comprehensive understanding of the potential role of K_hib_ in the growth, development, and physiological activities of soybean, a systematic analysis of K_hib_ modification in soybean leaves was performed in this study. Total proteins were extracted from soybean leaves at different growth stages and the K_hib_-modified proteins were then identified by three independent tests, including affinity enrichment using K_hib_ pan antibody and nano-HPLC–MS/MS analysis.

**Fig. 1:**
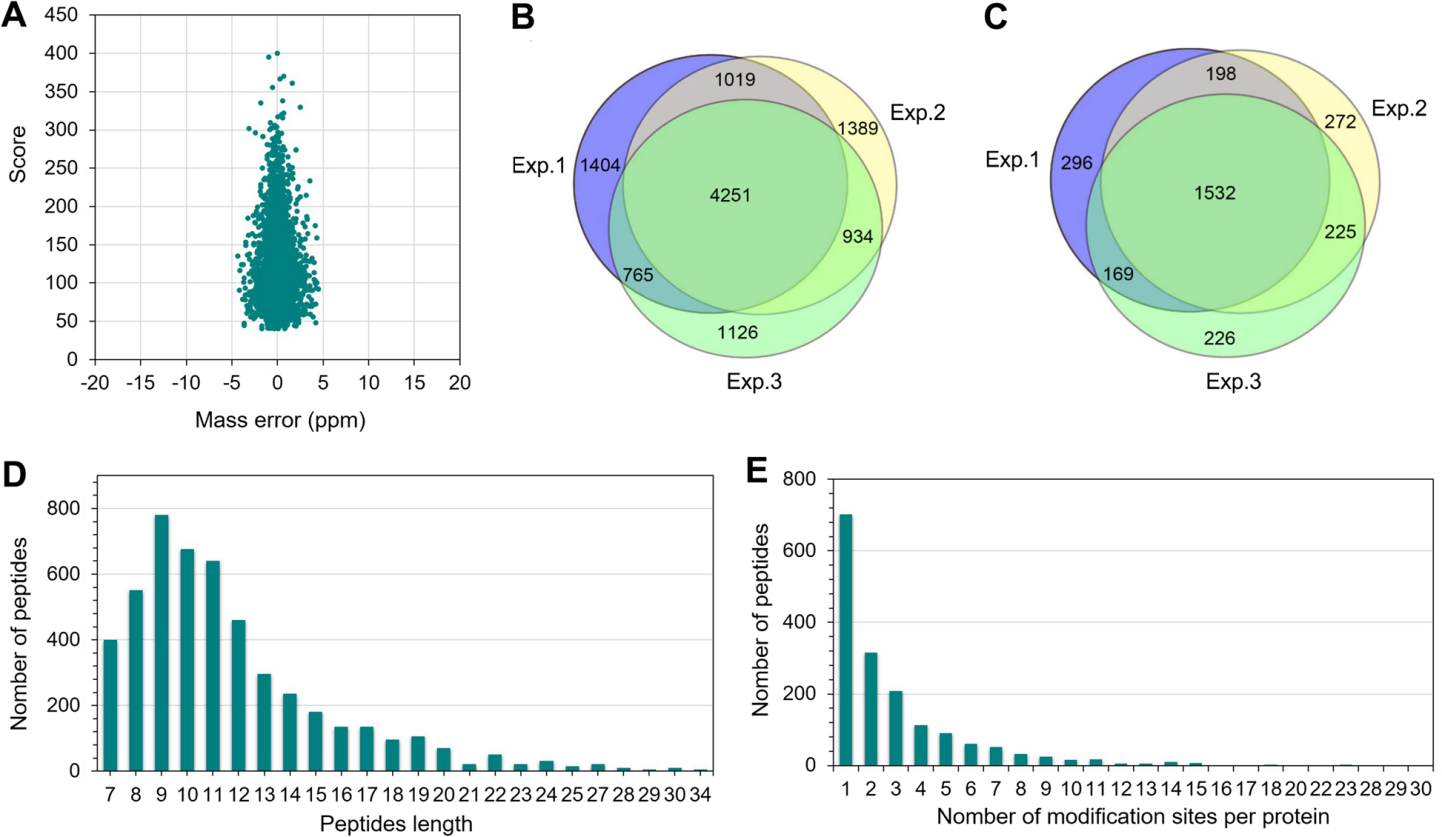
Global analysis of lysine 2-hydroxyisobutyrylation (K_hib_) peptides and proteins identified in soybean leaves. **A** Mass error and score distribution of all identified K_hib_-modified peptides. **B** Venn diagram shows the K_hib_ peptides identified in three experiments. **C** Venn diagram shows the K_hib_ proteins identified in three experiments. **D** Peptide length distribution of all K_hib_-modified peptides. **E** Protein distribution of K_hib_-modified sites contained in each protein

The identification results of the above mentioned three replicates are shown in Fig. 1 and Table S1. In total, 7287, 7439 and 6932 K_hib_-modified peptides were identified, respectively, of which 4251 peptides overlapped in three replicates (Fig. 1B), with peptide scores above 40 and mass errors within 5 ppm (Fig. 1A), indicating that highly accurate and high-quality MS data were obtained and that the data met the requirements for further analysis. The length distribution of K_hib_-modified peptides was analyzed and found to vary from 7 to 34 amino acid residues, but most of the peptides were between 7 and 20 residues in length (Fig. 1D).

Furthermore, the K_hib_-modified peptides identified in the three replicates matched 2195, 2227 and 2152 proteins, respectively, with 1532 proteins overlapping in the three replicates (Fig. 1C). The 1532 K_hib_-modified proteins contained a range of 1–30 K_hib_ sites per protein, of which 42.1% contained 1 K_hib_ site, 43.5% contained 2–5 K_hib_ sites, 11.1% contained 6–10 K_hib_ sites, and the remaining 3.3% contained more than10 K_hib_ sites (Fig. 1E), indicating that most of the proteins contained multiple K_hib_ sites. Surprisingly, several proteins contained more than 28 K_hib_ sites, such as lipoxygenase (accession number: A0A0R0G554, 28 sites), Glutamine amidotransferase type-2 domain-containing protein (accession number: A0A368UIE9, 30 sites) and an uncharacterized protein (accession number: I1MRV6, 29 sites) (Table S1). The distribution of K_hib_-modified peptide lengths and the number of K_hib_ sites contained in individual proteins in soybean were similar to those found in wheat [21, 22] and rice [20]. The identification of such a large number of K_hib_-modified sites and proteins in soybean not only indicates that K_hib_ modification is an abundant and complex PTM, but also indicates that this modification may have a very important role in regulating protein functions in soybean. In a word, we have characterized the lysine 2-hydroxyisobutyrylome in soybean for the first time, and the obtained data on K_hib_ modification will help to elucidate the potential regulatory role of K_hib_ in the physiological functions of soybean.

### Functional characterization, subcellular localization and secondary structure analysis of K_hib_-modified proteins in soybean

Considering that K_hib_-modified proteins may have important physiological roles in soybean, we performed Gene Ontology (GO) classification analysis to facilitate the understanding of the potential functions of K_hib_-modified proteins that identified in soybean. GO annotation is commonly used to determine the role of genes or proteins based on three independent ontologies, including biological process, molecular function and cellular component [31].

**Fig. 2:**
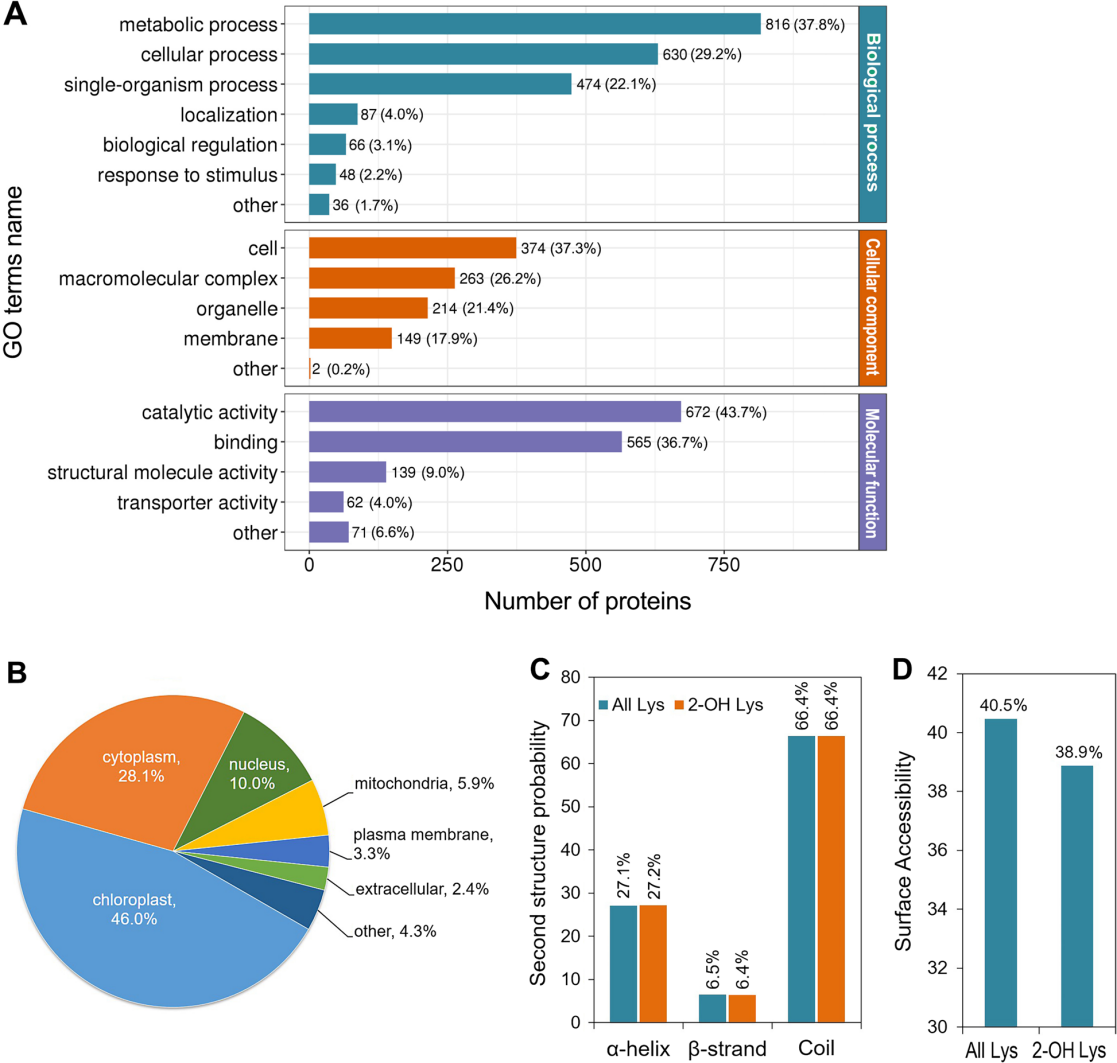
Functional classification, secondary structure and subcellular localization analysis of the K_hib_-modified proteins in soybean leaves. **A** Functional classification based on GO annotations. **B** Subcellular localization distribution of K_hib_-modified proteins. **C** Predicted secondary structure of K_hib_-modified peptides. **D** Predicted surface accessibility of K_hib_-modified peptides

The classification results were shown in Fig. 2A and Table S2, from which it is clear that K_hib_-modified proteins are involved in a variety of biological processes, molecular functions and cellular components. In the classification of “biological process”, most K_hib_-modified proteins were classified into three major categories: metabolic processes, cellular processes and individual organ processes, accounting for 37.8%, 29.2% and 22.0% of the total number of K_hib_-modified proteins, respectively (Fig. 2A, cyan bars). In the classification of “molecular function”, most K_hib_-modified proteins were classified into two categories, namely catalytic activity (43.7%) and binding (36.7%) (Fig. 2A, purple bars), which is in agreement with the classification of biological processes, and both results suggest that enzymatic proteins related to metabolism are more preferred to be K_hib_ modified in soybean. This finding suggests that K_hib_ modification may play an important role in metabolism and cellular process. As for the classification of “cellular component”, 37.3% of K_hib_ proteins were in the cell, 26.2% in the macromolecular complex, 21.4% in the organelle and 14.9% in the membrane (Fig. 2A, orange bars). The subcellular localization of K_hib_-modified proteins was also predicted using WoLF PSORT software, which showed that most of the proteins were distributed in chloroplasts (46.0%), cytoplasm (28.1%) and nucleus (10.0%) (Fig. 2B and Table S3).

In addition, we performed secondary structure analysis of the identified proteins to further assess the effect of K_hib_ modification on protein function. The results showed that 27.2% of all K_hib_ sites were located in the α-helix, 6.4% in the β-strand, and up to 66.4% in the coil (Fig. 2C and Table S4), indicating that K_hib_ has a preference for secondary structures. However, the distribution trend of K_hib_ sites was not significantly different from the distribution of all lysine sites (Fig. 2C). Moreover, we further assessed the surface accessibility of the identified K_hib_ sites and found that 38.9% of the K_hib_ sites were exposed on the protein surface, slightly lower than all lysine sites (40.5%) (Fig. 2D and Table S4). The lower surface accessibility of the K_hib_ sites indicates that K_hib_ modification may occur in a selective process, which is similar to the percentage of surface accessibility of K_hib_-modified proteins found in wheat [21]. Furthermore, the distribution trends of the functional classification, subcellular localization and secondary structure of K_hib_ proteins found in soybean are very similar to those of K_ac_-modified proteins in soybean [28] and also to those of K_hib_-modified proteins in other plants, such as wheat [5, 20, 22] and rhubarb [23]. Taken together, these findings suggest that protein K_hib_ modification may be a conserved mechanism for regulating protein function in plants.

### Enrichment analysis of K_hib_-modified proteins in soybean

**Fig. 3:**
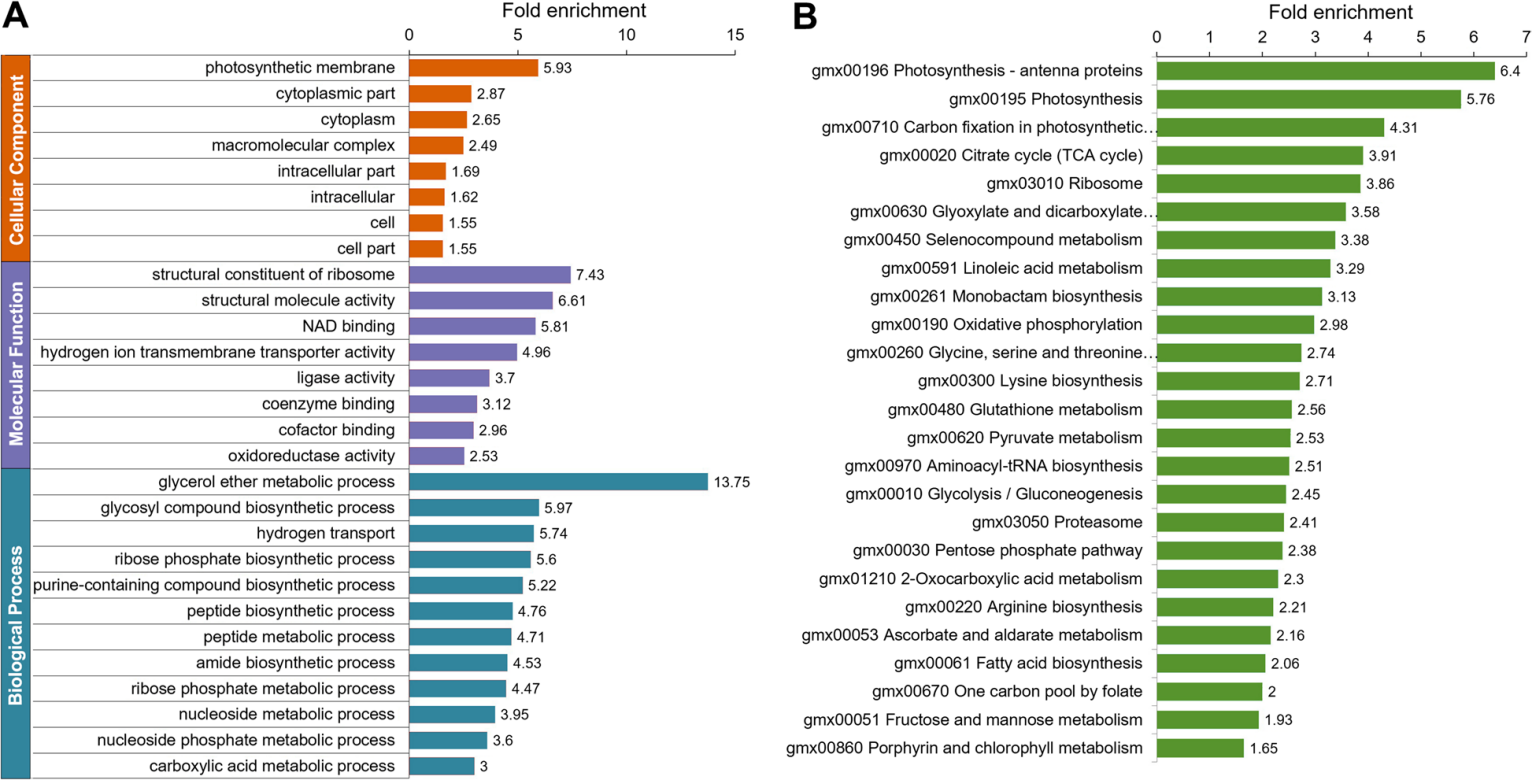
Enrichment analysis of the K_hib_-modified proteins in soybean leaves. **A** GO enrichment in terms of biological process, cellular component and molecular function. **B** KEGG pathway enrichment. The x-axis represents fold enrichment and the y-axis represents the categories of GO terms

To further characterize the nature of K_hib_-modified proteins in soybean, we performed enrichment analysis both of GO annotation and KEGG pathways for the K_hib_-modified proteins identified in this study. As shown in Fig. 3 and Table S5, the K_hib_-modified proteins were found to be significantly enriched in both the GO annotation and KEGG pathway.

In the enrichment of GO molecular function, structural constituent of ribosome, structural molecule activity, NAD binding, hydrogen ion transmembrane transporter activity, and ligase activity, coenzyme binding, cofactor binding and oxidoreductase activity, etc., were found to be significantly enriched (Fig. 3A, purple bars, and Fig. S1). The enrichment of these molecule functions indicates that those proteins whose functions are related to ribosome and metabolism may prefer to be K_hib_ modified. The enrichment result of GO biological process showed that glycerol ether metabolic process, glycosyl compound biosynthetic process hydrogen transport, ribose phosphate biosynthetic process, purine-containing compound biosynthetic process were the top 5 highly enriched biological processes (Fig. 3A, cyan bars, and Fig. S1). These five enriched biological processes and the remaining significantly enriched biological processes shown in Fig. 3A are mainly related to metabolism and biosynthesis, which is consistent with the enrichment of GO molecule functions, suggesting that K_hib_ modification is more important for the regulation of biosynthesis and metabolism than on other physiological functions. For the enrichment analysis of GO cellular component, the K_hib_-modified proteins were significantly enriched in photosynthetic membrane, cytoplasmic part, cytoplasm, and macromolecular complex, etc. (Fig. 3A, orange bars, and Fig. S1).

**Fig. 4:**
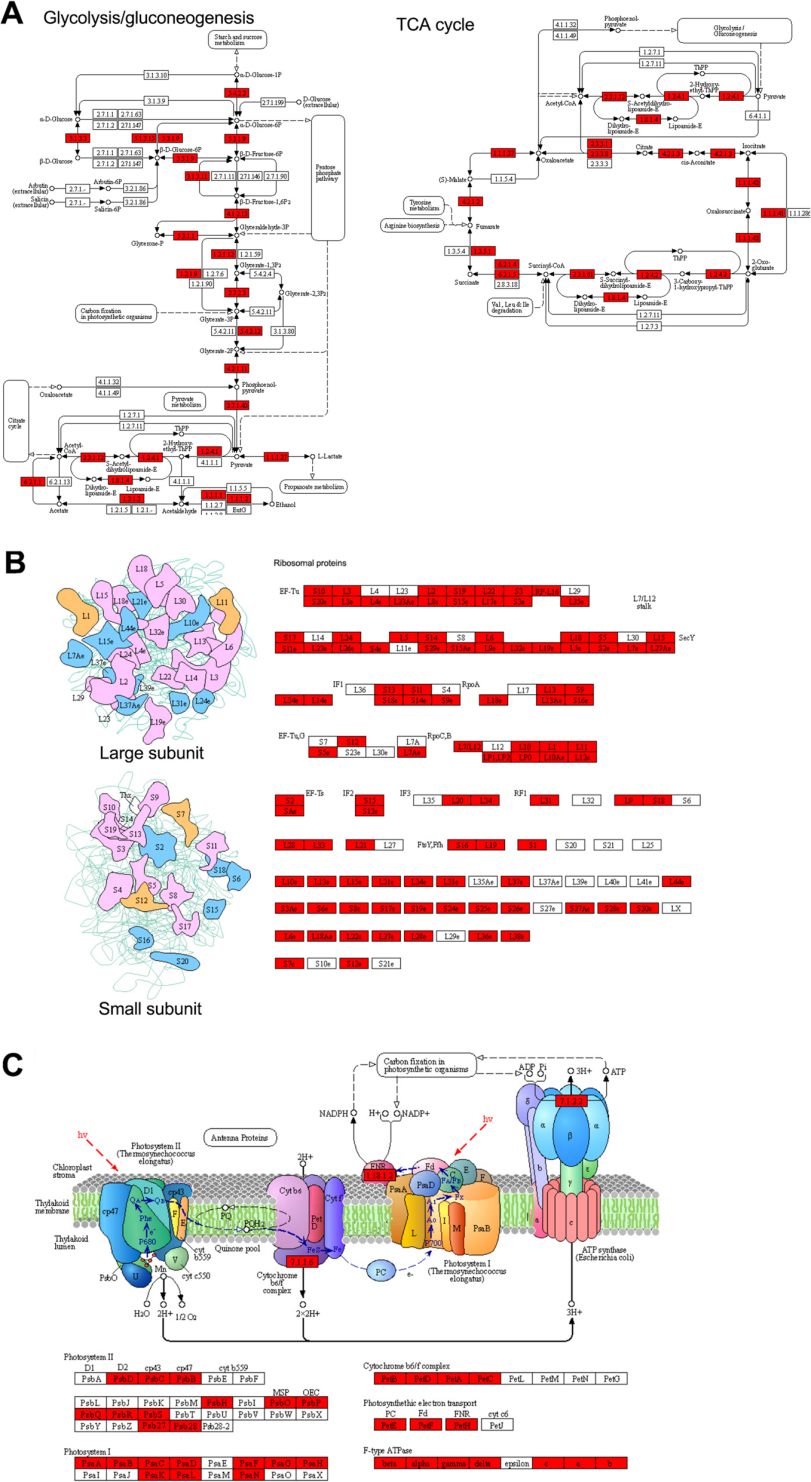
Representative KEGG pathways showing significant enrichment of K_hib_-modified proteins in soybean leaves. **A** Central metabolism, including glycolysis/gluconeogenesis, pentose phosphate pathway, TCA cycle and oxidative phosphorylation. **B** Ribosome. The identified K_hib_ enzymes are indicated with a red background

Consistent with the GO enrichments, a number of pathways related to metabolism and biosynthesis were found to be significantly enriched in KEGG pathway analysis (Fig. 3B, Fig. S2 and Table S6). Among these enriched pathways, citrate cycle (TCA cycle), glyoxylate and dicarboxylate metabolism, oxidative phosphorylation, glycine, serine and threonine metabolism, pyruvate metabolism, glycolysis/gluconeogenesis, pentose phosphate pathway, and fructose and mannose metabolism are involved in central carbon metabolism (the K_hib_-modified proteins in glycolysis/gluconeogenesis and TCA is shown in Fig. 4A, K_hib_-modified proteins in pentose phosphate pathway and oxidative phosphorylation shown in Fig. S3A), and pathways of ribosome, selenocompound metabolism, monobactam biosynthesis, glycine, serine and threonine metabolism, lysine biosynthesis, glutathione metabolism, aminoacyl-tRNA biosynthesis, and arginine biosynthesis are involved in biosynthesis (the K_hib_ proteins in ribosome shown in Fig. 4B). These findings also indicated that proteins related to metabolism and biosynthesis tended to be K_hib_ modified. Moreover, several pathways involved in photosynthesis and fatty acid metabolism were also found to be markedly enriched (the K_hib_ proteins in photosynthesis shown in Fig. 4C, and the K_hib_ proteins in carbon fixation in photosynthetic organisms and fatty acid biosynthesis shown in Fig. S3B and C), suggesting that photosynthesis and fatty acid metabolism in soybean may also be regulated by K_hib_ modification. In addition, the pathways associated with proteasome, 2-oxocarboxylic acid metabolism, ascorbate and aldarate metabolism, and porphyrin and chlorophyll metabolism were also found to be significantly enriched (Fig. 3B).

Taken together, the enrichment analysis results suggest that K_hib_-modified proteins related to biosynthesis and metabolism are extremely important for the physiological functions of soybean leaves. Notably, K_hib_-modified proteins involved in metabolism and biosynthesis were also found to be significantly enriched in other plants, such as rice [19, 20], wheat [5, 22], and rhubarb [23]. The enrichment of K_hib_-modified proteins associated with central carbon metabolism and biosynthesis found in plants for which global analyses of K_hib_ modifications have been performed to date suggests that K_hib_ modifications of these proteins may be the most important regulation pattern in plants. Furthermore, K_hib_-modified proteins enriched in photosynthesis were found not only in soybean in this study, but also in wheat [21, 22] and rice [20]. As we all known that photosynthesis is a fundamental biological process involving the conversion of solar energy into chemical energy, as well as a primary producer of oxygen, which is crucial for plants and even all life on Earth [32]. In fact, photosynthesis-related pathways including photosynthesis-antenna proteins, photosynthesis and carbon fixation in photosynthesis organisms were the top 3 enriched KEGG pathways found in this study (Fig. 3B), indicating that K_hib_ modification is also important for photosynthesis in plants. Therefore, the discovery of K_hib_ modification of photosynthesis-related proteins will help to fully elucidate the mechanism of photosynthesis of leaves.

### Analysis of K_hib_ site properties reveals conserved motifs in soybean

**Fig. 5:**
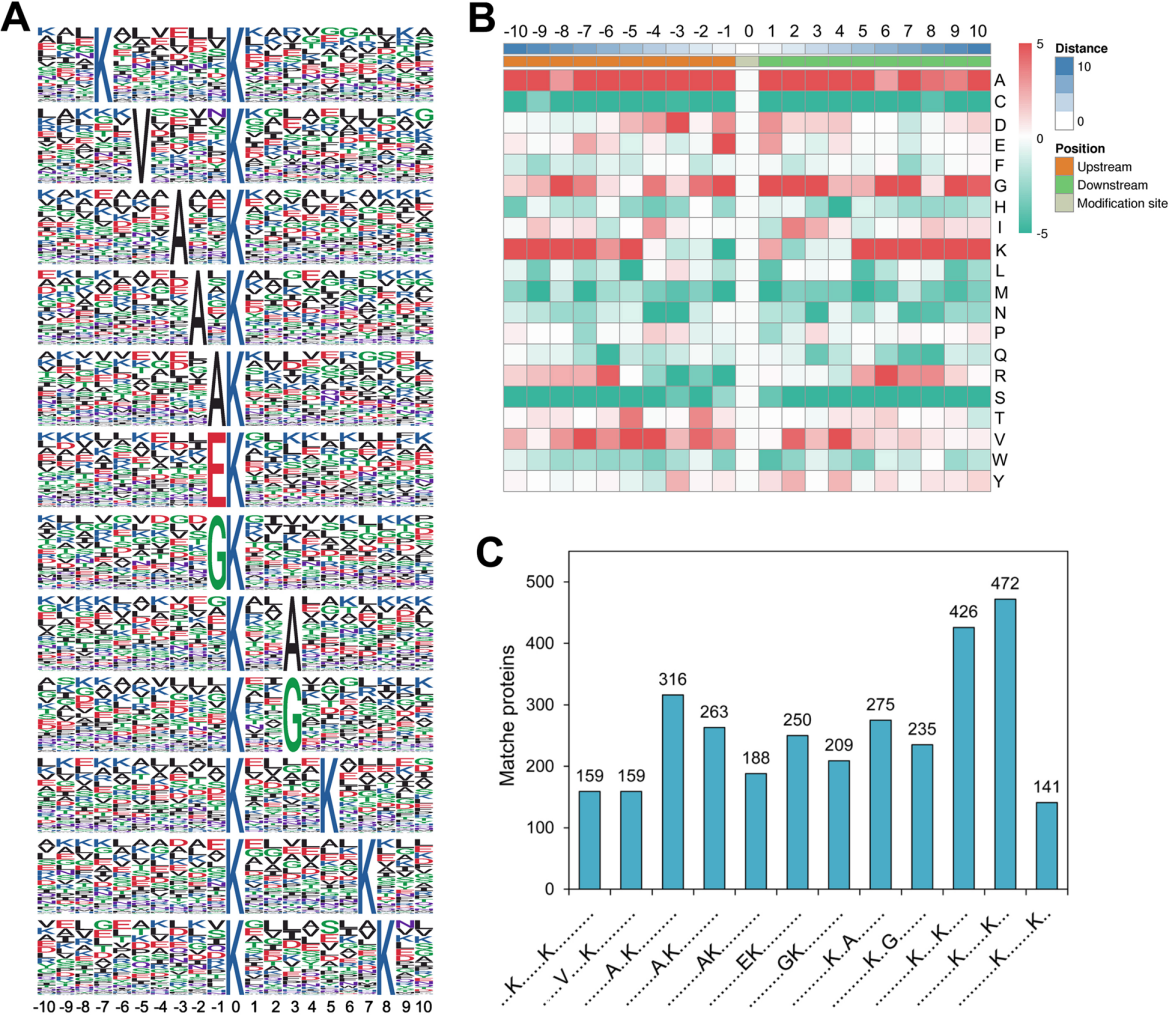
Motif analysis of the identified K_hib _peptides in soybean leaves. **A** The sequence motifs of amino acids from − 10 to + 10 flanking the K_hib _sites. The size of the letter represents the frequency of the amino acid residue residing at that position (**B**) Heat map analysis of the frequency of amino acid residues residing from -10 to + 10 positions around the K_hib _sites. The enrichment or dispersion of amino acids are shown in red or green, respectively. **C** The histogram shows the number of K_hib_-modified peptides matched for each motif.

To further analyze the natural properties of K_hib_-modified proteins in soybean, we investigated the sequence motifs around the K_hib_ sites of all identified K_hib_-modified peptides using the Motif-X software, a tool for extracting overrepresented patterns [33]. In total, 12 conserved motifs were obtained with amino acid residues ranging from -10 to + 10 surrounding the K_hib_ site, such as K*K_hib_, K_hib_*K, V*K_hib_, A*K_hib_, K_hib_*A, EK_hib_, GK_hib_ and K_hib_*G (“*” indicates one or more random amino acid residues, Fig. 5A and Table S7). Investigation of these motifs found that only five residues are preferred flanking the K_hib _site, including a basic amino acid residue (K), an acidic amino acid residue (E), and three aliphatic amino acid residues with small side chains (G, A and V). Thus, based on the different properties of these five amino acid residues, these 12 motifs could be divided into three patterns: + 5 to + 8 (except + 6) or -7 position for a basic amino acid residue (K……K_hib_….K.KK), -1 position for an acidic amino acid residue (EK_hib_), and -1 to -5 (except -4) or + 3 position for an aliphatic amino acid residue with a small side chain (V.AAAK_hib_.A/G) (“.” indicates a random amino acid residue at that position).

Consistent with the results of motif analysis, the heat map of the frequency of occurrence of amino acid residues flanking the K_hib _site showed that A, G, K and V at several positions were overrepresented around the K_hib _site (Fig. 5B). However, C, M and S were significantly underrepresented at all positions from -10 to + 10 flanking the K_hib _site. Among these motifs, the most distributed motifs are K_hib_…..K and K_hib_….K, matching to 472 (15.3%) and 426 (13.8%) K_hib _peptides, respectively (Fig. 5C). However, due to the other two motifs flanked by K (K…..K_hib _and K_hib_……K) match fewer peptides, the third type of motif pattern, namely K_hib _flanked by G, A and V, matched the largest number of K_hib _peptides among the three types of motif patterns mentioned above, accounting for 53.2% of all matched peptides (Fig. 5C).

The motif pattern of K_hib _site flanking by K at several positions is highly similar to the sequence motifs obtained in other plants, such as wheat [22] and rhubarb [23]. Additionally, although motifs with K flanking the K_hib _site were also found in rice, K only occurs at the position of + 9 or -1 [20]. Unlike the motif pattern of K_hib _site flanking by K, the motif pattern of K_hib _site flanking by E, namely EK_hib_, was found in rhubarb [23], rice [20] and *Arabidopsis siliques*[7], but not in wheat. In the third motif pattern mentioned above, with the exception of GK_hib _found in rhubarb [23], the motifs of K_hib _site flanked by A or G at different positions is only found in *Arabidopsis siliques*[7], while the motif of K_hib _site flanked by V is currently not found in other plants and is unique to soybean.

The several types of motif patterns extracted from plants suggest that there may be multiple enzymes responsible for catalyzing protein K_hib _modification in plants. Furthermore, the above comparative analysis of motifs in different plants showed both similarities and differences in the motifs extracted in different plants, which seems to suggest that there may be similar enzymes to catalyze K_hib _modifications of proteins in different plants, but there may also be specific enzymes for each plant.

### Analysis of protein interaction networks of K_hib_-modified proteins in soybean

**Fig. 6:**
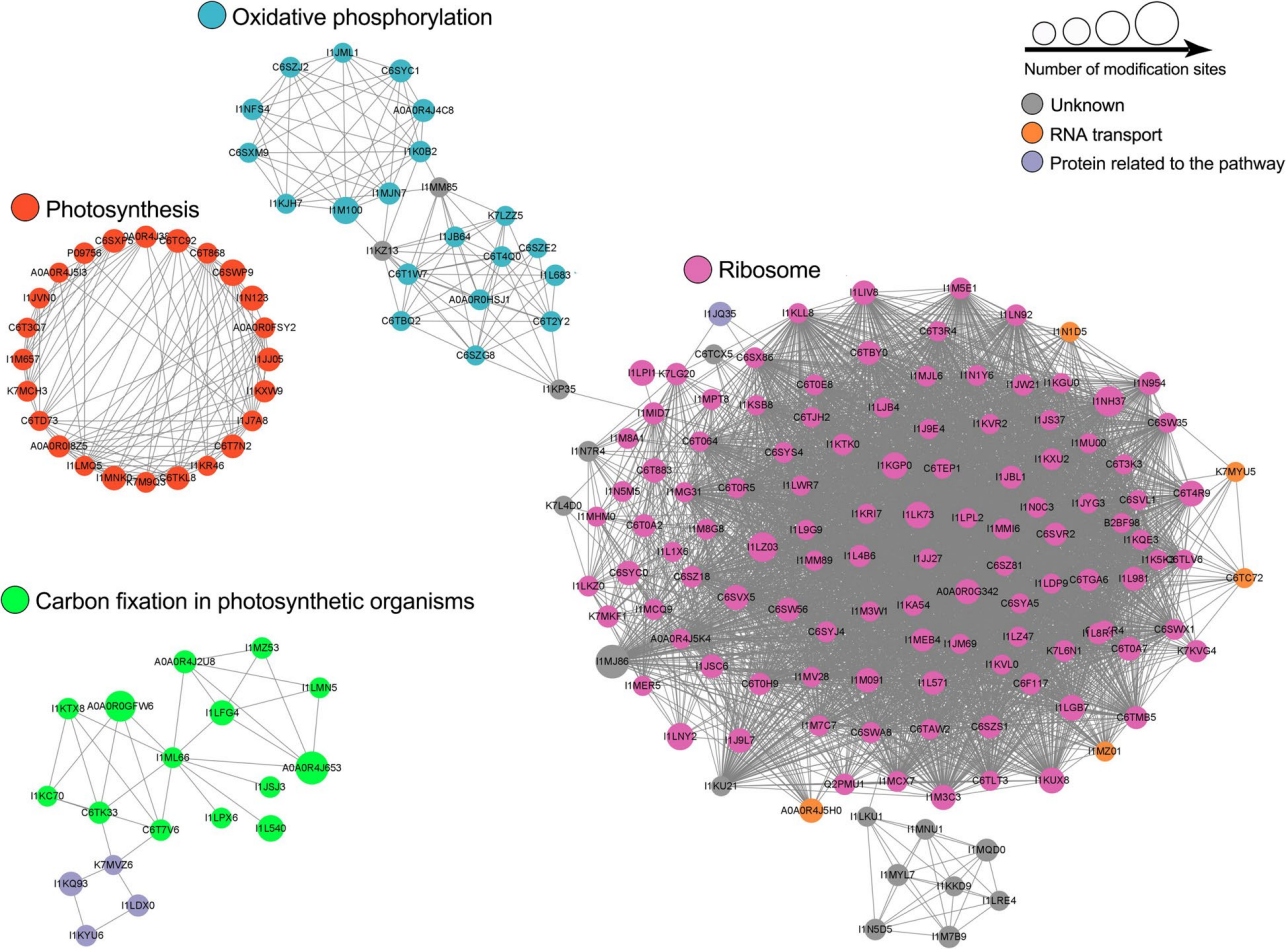
PPI network analysis of the K_hib_-modified proteins in soybean leaves. Four representatives of PPI subnetworks, including ribosome, oxidative phosphorylation, photosynthesis and carbon fixation in photosynthetic organisms are presented

To better understand the cellular processes regulated by K_hib _modification in soybean, we performed a protein–protein interaction network for all of the K_hib_-modified proteins using the STRING database and Cytoscape software [34]. The results showed that a total of 1043 K_hib_-modified proteins were matched as the network nodes and 5501 interactions were obtained from the STRING database with a combined score ≥ 0.90. The global PPI network graph of these interactions is shown in Fig. S4, and the detailed interactions are shown in Table S8. In addition, 16 clusters of K_hib _proteins were retrieved from the global PPI network by using a clustering algorithm performed with the MCODE plug-in tool kit (Table S8), and 5 representatives of highly-connected clusters, including ribosome, photosynthesis, oxidative phosphorylation, carbon fixation in photosynthetic organisms and TCA cycle are shown in Fig. 6. These data suggest that K_hib _modifications tend to occur in proteins associated with specific functional clusters. Notably, these extracted clusters are consistent with both KEGG pathway and GO annotation enrichment analysis.

Prior to the present study, the complete interaction network of K_hib_-modified proteins in plants was identified only in rice flowers [29] and seedlings [20]. Ribosomes, photosynthesis, and carbon metabolism were identified as abundant clusters in rice seedlings [20], and these clusters differ from the highly connected clusters identified in rice flowers which include ribosome, proteases, ATPases, heat shock protein 70 (HSP70), histones, mitochondrial proteins, TCA cycle and glycolytic enzymes [29]. The similar PPI clusters, such as ribosomes, photosynthesis, TCA cycle and oxidation phosphorylation were obtained in this study, suggesting that K_hib _modification has an important regulatory role in these physiological processes in plants.

## Conclusions

In the present study, the first systematic analysis of K_hib_-modified proteins in soybean, an important oil crop and industrial crop, was performed by using a highly sensitive proteomic method. A total of 4251 overlapping K_hib_-modified peptides matched to 1532 proteins were identified in three replicates, which showed diverse subcellular compartments distribution. Functional and pathway analysis of the K_hib_-modified proteins suggested that these proteins are involved in multiple cellular functions and metabolic pathways. Moreover, K_hib _modifications were found to occur preferentially in proteins associated with ribosome/biosynthesis, central carbon metabolism, photosynthesis and fatty acid biosynthesis, suggesting that K_hib _modifications play a particularly important regulatory role in these pathways in soybean leaves. Furthermore, three types of motif patterns extracted from 12 K_hib _sequence motifs showed that A/V/G, E, and K were strongly biased around the K_hib _site. Our global analysis of K_hib_-modified proteins in soybean indicates that K_hib _is an abundant and conserved PTM in plants, and the obtained K_hib _proteome data will contribute to a better understanding of the potential physiological activity of K_hib _modifications in soybean. Although the above findings were obtained in this study, quantitative analysis of K_hib_-modified proteins and further biological experiments are needed to confirm the role of the identified K_hib _modifications in soybean.

## Materials and methods

### Plant material and cultivation

The soybean used in this study was cultivar Qihuang 34, and the cultivation of soybean was carried out at the experimental site of Shandong Agricultural University, as described previously [28]. Briefly, soybean seeds were sowed in the field and the type soil is Typic-Hapli-Udic Argosols according to the Chinese Soil Taxonomy [35]. Inverted trifoliate leaves were collected from three soybean plants of similar growth at early blooming flowering (R1), early podding (R3), early bulging (R5), and early maturity (R7), respectively, and kept in liquid nitrogen for 24 h and then stored at -80 °C. The collection of plant specimens has been approved by College of Agronomy, Shandong Agricultural University. Experimental research and field study on soybean in this study has complied with the IUCN Policy Statement on Research Involving Species at Risk of Extinction.

### Total protein extraction and trypsin digestion

The soybean leaves collected at different stages were mixed in equal amounts and used as biological replicates. Whole proteins of leaves were extracted by using a phenol isolation method [36] with a slight modification. Briefly, the 400 mg of leaves were grinded into powder in liquid nitrogen and 10% polyvinylpolypyrrolidone (PVPP) and then suspended in 1.6 mL lysis buffer 1 (1 M sucrose, 0.5 M Tris–HCl pH 8.0, 0.1 M KCl, 50 mM ascorbic acid, 1% NP40, 1% sodium deoxycolate, 10 mM EDTA, 10 mM DTT, and 1% plant protease inhibitor cocktail). The obtained sample solutions were sonicated on ice water and then placed on ice for 30 min. The resulting solutions were mixed with equal volumes of tris-saturated phenol (pH 8.0) and then placed on ice for 10 min, followed by centrifugation at 16,000 g for 10 min at 4 °C and collection of the upper phenol phase. The resulting phenol solutions were mixed with four volumes of precipitation buffer (methanol with 0.1 M ammonium acetate, precooled at -20 ℃ before use), and then placed overnight at -20 °C to precipitate the proteins. The precipitates were collected by centrifugation at 16,000 g for 10 min at 4 °C, and the pellets were rinsed twice with cold methanol. The protein pellets were dissolved in 1.6 mL lysis buffer 2 (8 M urea, 50 mM Tris–HCl pH 8.0, 1% NP40, 1% sodium deoxycolate, 10 mM EDTA, 5 mM DTT, 1% plant protease inhibitor cocktail and 1% phosphatase inhibitor cocktail), followed by sonication on ice water and centrifugation at 20,000 g for 10 min at 4 °C. The final supernatants were collected and the protein concentration was determined using the 2D Quant kit (GE Healthcare).

For tryptic digestion, 2 mg of proteins were first reduced with 5 mM DTT at 56 °C for 30 min, followed by alkylation with 30 mM iodoacetamide (IAM) at room temperature in the dark for 45 min. Then, proteins were precipitated by adding 5 volumes of -20 °C precooled methanol and placed at -20 °C overnight, followed by centrifugation at 20,000 g for 10 min at 4 °C, and the obtained precipitates were rinsed twice with precooled methanol and then placed at -20 °C for 1 h. Finally, the resulting proteins were suspended in 0.1 M triethylammonium bicarbonate (TEAB) buffer and digested overnight with 50 µg of trypsin (Promega). After digestion, the tryptic peptides were desalted using a Strata X C_18_SPE column (Phenomenex) and vacuum dried before affinity enrichment.

### Affinity enrichment of K_hib_-modified peptides

For affinity enrichment of K_hib_-modified peptides, the dried peptides were dissolved in NETN buffer (100 mM NaCl, 1 mM EDTA, 50 mM tris–HCl pH 8.0, 0.5% NP-40) and then incubated with anti-2-hydroxyisobutyryllysine pan antibody agarose beads (Micrometer Biotech) at 4 °C overnight with gentle shaking. Then, after washing the agarose beads four times with NETN buffer and once with double distilled water, the K_hib_-modified peptides were eluted with 0.1% trifluoroacetic acid (TFA) and desalted with a C18 ZipTips column, followed by vacuum drying for further use.

### Liquid chromatography-mass spectrometry analysis

The dried K_hib_-modified peptides were firstly dissolved in buffer A (0.1% formic acid (FA)) and then separated by liquid chromatography (LC) with a reversed-phase C_18_analytical column (Thermo Acclaim PepMap RSLC C_18_column, 75 μm × 500 mm, 2 μm particles) at a flow rate of 250 nL/min on an UltiMate RSLCnano 3000 system (Thermo Scientific) as described previously [37, 38]. The gradient was set to 2–10% buffer B (0.1% FA and 80% acetonitrile in water) for 6 min, 10–20% buffer B for 45 min, 20–80% buffer B for 7 min and finally hold at 80% for 4 min.

The eluted peptides were ionized and electrosprayed (2.0 kV voltage) into the mass spectrometer (Thermo Scientific Q Exactive HFX) coupled online to the LC system. The mass spectrometric analysis was performed in data-dependent mode with an automatic switch between a full MS scan and a MS/MS scan in the Orbitrap. The peptides were detected in the MS at a resolution of 60,000 with a scan range of 350–1800 m/z, and with automatic gain control (AGC) of 1E6. The top 15 precursor ions were selected for MS/MS by higher-energy collision dissociation (HCD) using NCE setting as 26%, and the MS/MS spectra were detected at a resolution of 30,000. The dynamic exclusion for the data-dependent scan was 6 s, and AGC was set at 1E5. LC–MS/MS analysis was performed by Micrometer Biotech Company (Hangzhou, China).

## Data analysis

The resulting MS/MS raw data were searched using MaxQuant search engine (v.1.5.2.8) against *Glycine max* (soybean) database from Uniprot (74,863 proteins, https://www.uniprot.org/proteomes/UP000008827). False discovery rate (FDR) thresholds for peptides, proteins and modifications adjusted to < 1%. Trypsin/P was specified as a cleavage enzyme with up to 4 missing cleavages and set the minimum number of amino acids for peptide as 7. Carbamidomethylation on Cys was specified as a fixed modification and oxidation on Met, 2-hydroxyisobutyrylation on Lys were specified as variable modifications. The mass tolerances for precursor ions and fragment ions were set at 10 ppm and 0.02 Da, respectively. K_hib _sites localization probability was set as > 0.75, minimum score for modified peptides was set > 40 and minimum delta score for modified peptides was set > 8. The identification of modified peptides requires at least 1 MS/MS. The MS data were deposited to ProteomeXchange Consortium via the PRIDE partner repository. The accession number is PXD03650.

### Functional annotation of K_hib_-modified peptides and proteins

To explore the potential roles of the identified K_hib_**-**modified peptides and proteins, detailed bioinformatics analyses were performed. Functional annotation of Gene Ontology (GO) was performed for the functional classification and functional enrichment analysis [39]. For functional annotation of Kyoto Encyclopedia of Genes and Genomes (KEGG), the KAAS tool (v.2.0) was used for pathway enrichment analysis [40]. GO term or KEGG pathway enrichment analysis were carried out with the DAVID tool [41], and annotation terms with a corrected p-value < 0.05 by Fisher’s exact test were considered significantly enriched. The motif-X software [33] was used to analyse amino acid sequence motifs of K_hib _peptides. Motif-based clustering analysis was also performed and heatmap visualization of cluster members was performed using the “heatmap.2” function in the “gplots” R package. Subcellular location analysis of identified proteins was conducted using the WoLF PSORT platform (https://wolfpsort.hgc.jp). Secondary structure and surface accessibility analysis were performed using NetSurfP online software (v.2.0). STRING (v.11.0) [31] was used to evaluate potential protein–protein interaction relationships among those identified proteins, and only confidence score ≥ 0.7 were selected as significant. PPI networks were constructed and visualized using Cytoscape software (v.3.7) [42], and modules of PPI networks were screened using the Molecular Complex Detection (MCODE) plug-in tool in Cytoscape.

## Supplementary Information

**Additional file 1: Fig. S1.** The bubble chart shows the GO enrichment analysesof K_hib_-modifiedproteins in soybean leaves. **Fig. S2. **Thebubble chart shows the enrichment of KEGG pathway of K_hib_-modifiedproteins in soybean leaves. **Fig. S3. **Representative pathway mapsof K_hib_-modified proteins. (A) K_hib_-modified proteins in central carbon metabolism, includingpentose phosphate pathway and oxidative phosphorylation. (B) K_hib_-modifiedproteins in carbon fixation in photosynthetic organisms. (C) K_hib_-modifiedproteins in fatty acid biosynthesis. The identified K_hib_-modifiedproteins are indicated in boxes with a red background. **Fig. S4. **The global PPI network ofidentified K_hib_-modified proteins in soybean leaves. **Table S1.** All identified Khib sites and proteins that overlap in three replicates in soybean leaves. **Table S2.** GO functional classification of K_hib_-modified proteins in soybean leaves. **Table S3.** Subcellular localization distribution of K_hib_-modified proteins in soybean leaves. **Table S4.** Secondary structure and surface accessibility distribution of K_hib_-modified peptides in soybean leaves. **Table S5.** GO enrichment analysis of K_hib_-modified proteins in soybean leaves. **Table S6.** KEGG pathway enrichment of K_hib_-modified proteins in soybean leaves. **Table S7**. Motif analysis of K_hib_-modified peptides in soybean leaves. **Table S8.** The PPI network of K_hib_-modified proteins in soybean leaves.

## Data Availability

The raw data of MS spectrometry were deposited to the ProteomeXchange Consortium via the PRIDE partner repository with the dataset identifier of PXD036505 (http://www.ebi.ac.uk/pride/archive/projects/PXD036505).
