# Supplementary material for: Proteomic analysis of protein lysine 2-hydroxyisobutyrylation (Khib) in soybean leaves
Source: BMC Plant Biol. 2023 Jan 12;23:23. doi: 10.1186/s12870-022-04033-6 (PMC9835227; doi:10.1186/s12870-022-04033-6)
Supplement: Supplementary file 1 — Additional file 1: Fig. S1. The bubble chart shows the GO enrichment analysesof Khib-modifiedproteins in soybean leaves. Fig. S2. Thebubble chart shows the enrichment of KEGG pathway of Khib-modifiedproteins in soybean leaves. Fig. S3. Representative pathway mapsof Khib-modified proteins. (A) Khib-modified proteins in central carbon metabolism, includingpentose phosphate pathway and oxidative phosphorylation. (B) Khib-modifiedproteins in carbon fixation in photosynthetic organisms. (C) Khib-modifiedproteins in fatty acid biosynthesis. The identified Khib-modifiedproteins are indicated in boxes with a red background. Fig. S4. The global PPI network ofidentified Khib-modified proteins in soybean leaves. Table S1. All identified Khib sites and proteins that overlap in three replicates in soybean leaves. Table S2. GO functional classification of Khib-modified proteins in soybean leaves. Table S3. Subcellular localization distribution of Khib-modified proteins in soybean leaves. Table S4. Secondary structure and surface accessibility distribution of Khib-modified peptides in soybean leaves. Table S5. GO enrichment analysis of Khib-modified proteins in soybean leaves. Table S6. KEGG pathway enrichment of Khib-modified proteins in soybean leaves. Table S7. Motif analysis of Khib-modified peptides in soybean leaves. Table S8. The PPI network of Khib-modified proteins in soybean leaves. [file 12870_2022_4033_MOESM1_ESM.zip › Supportting information-Supplemental Figures and Figure Legends.docx]

**Supplementary information**

**Proteomic analysis of Protein Lysine 2-Hydroxyisobutyrylation (K_hib_) in soybean leaves**

Wei Zhao^1#^, Ting-Hu Ren^1#^, Yan-Zheng Zhou^2^, Sheng-Bo Liu^1^, Xin-Yang Huang^2^, Tang-Yuan Ning^1*^, Geng Li^1*^

^1^ College of Agronomy, Shandong Agricultural University, Tai’an, Shandong 271018, P. R. China

^2^ Jining Academy of Agricultural Sciences, Jining, Shandong 272075, P. R. China

***Corresponding author:**

Geng Li, E-mail: ligeng213@sina.com

Tang-Yuan Ning, E-mail: ningty@163.com

**^#^ These authors contribute equally to this work：**

Wei Zhao, E-mail: zhaowei9298@163.com

Ting-Hu Ren, E-mail: [rthu112233@163.com](mailto:rthu112233@163.com)

**Contents**

1. Supplemental Figures and Figure Legends

2. Supplemental Tables

**1. Supplemental figures and figure legends**


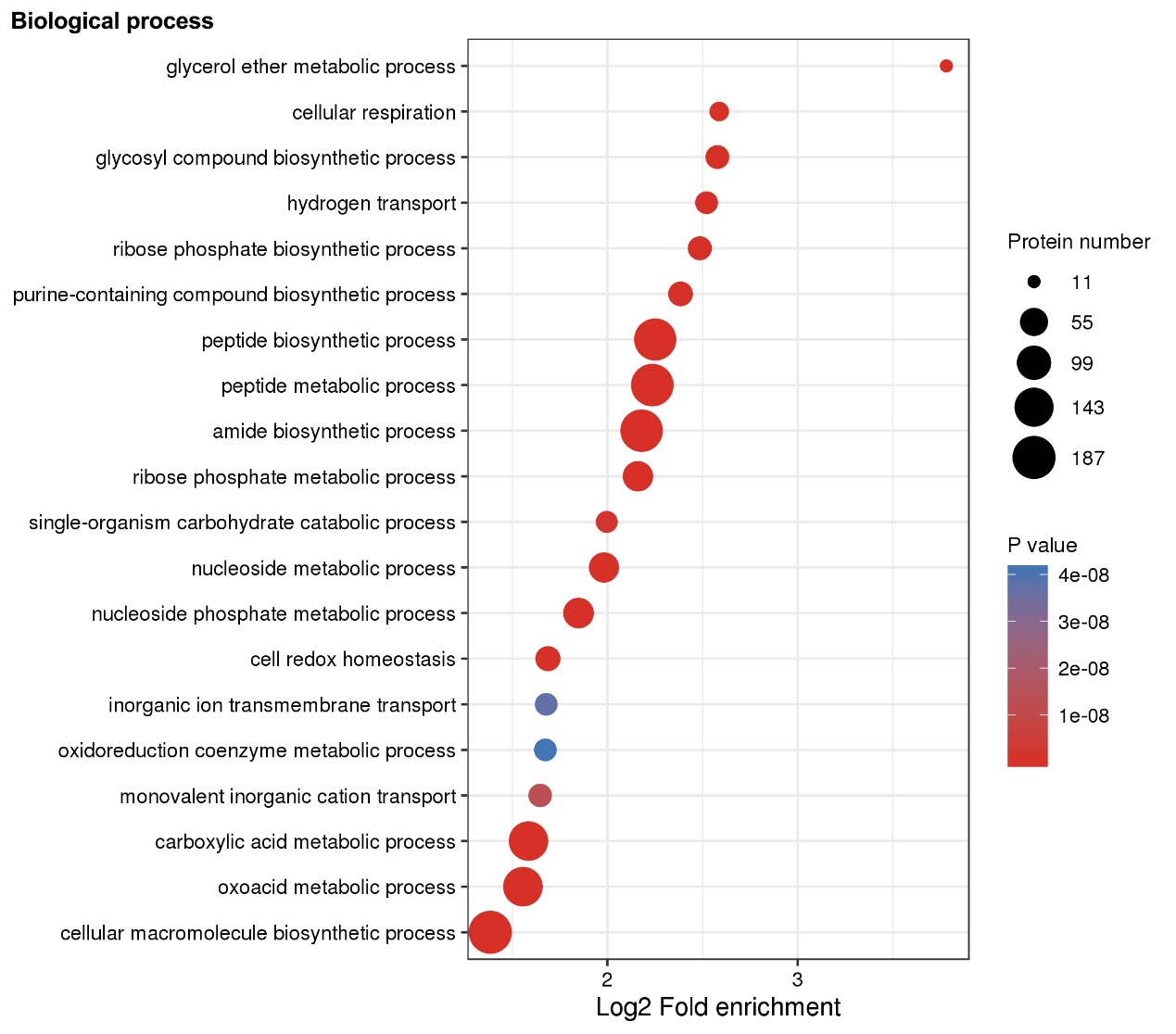


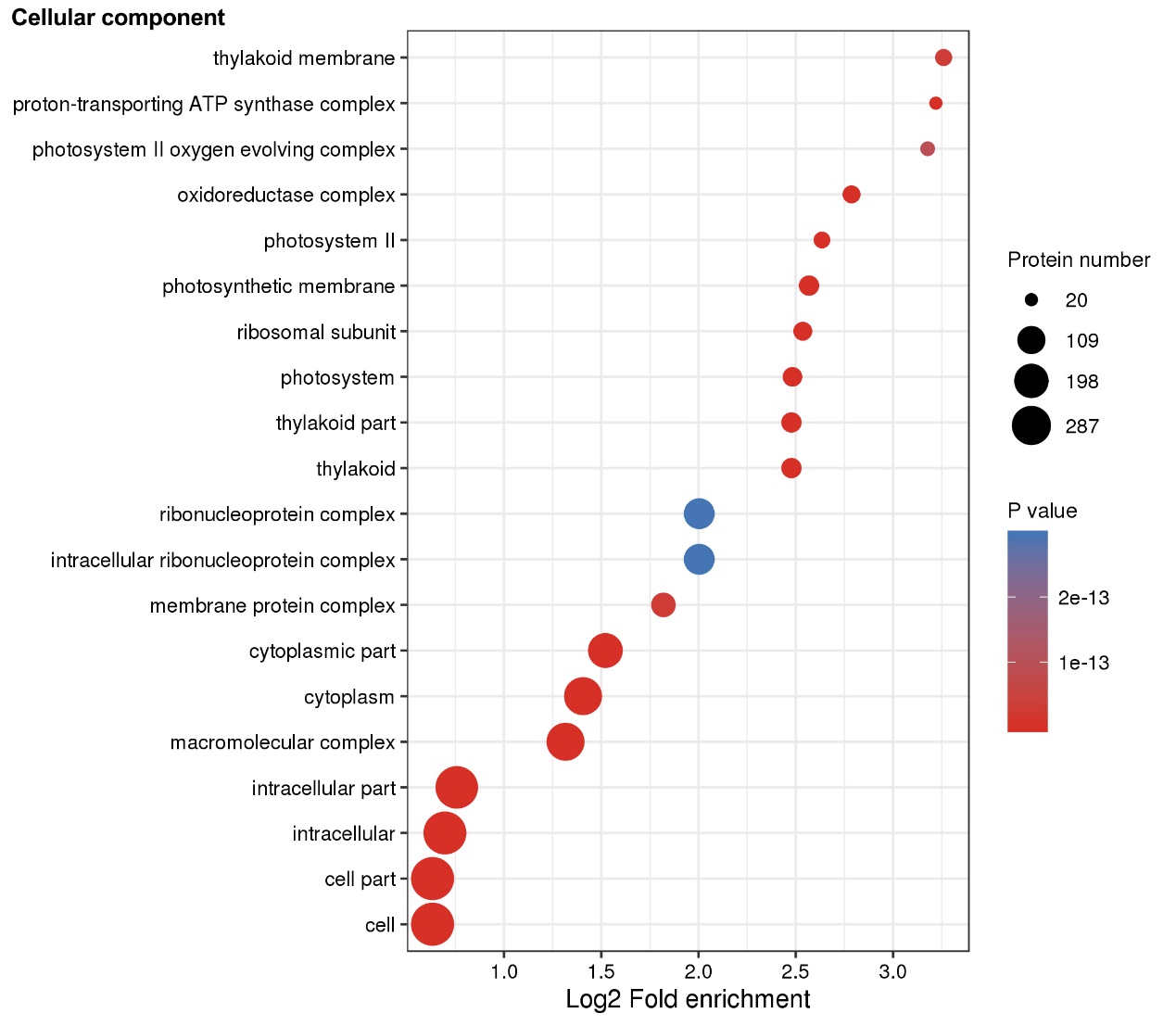


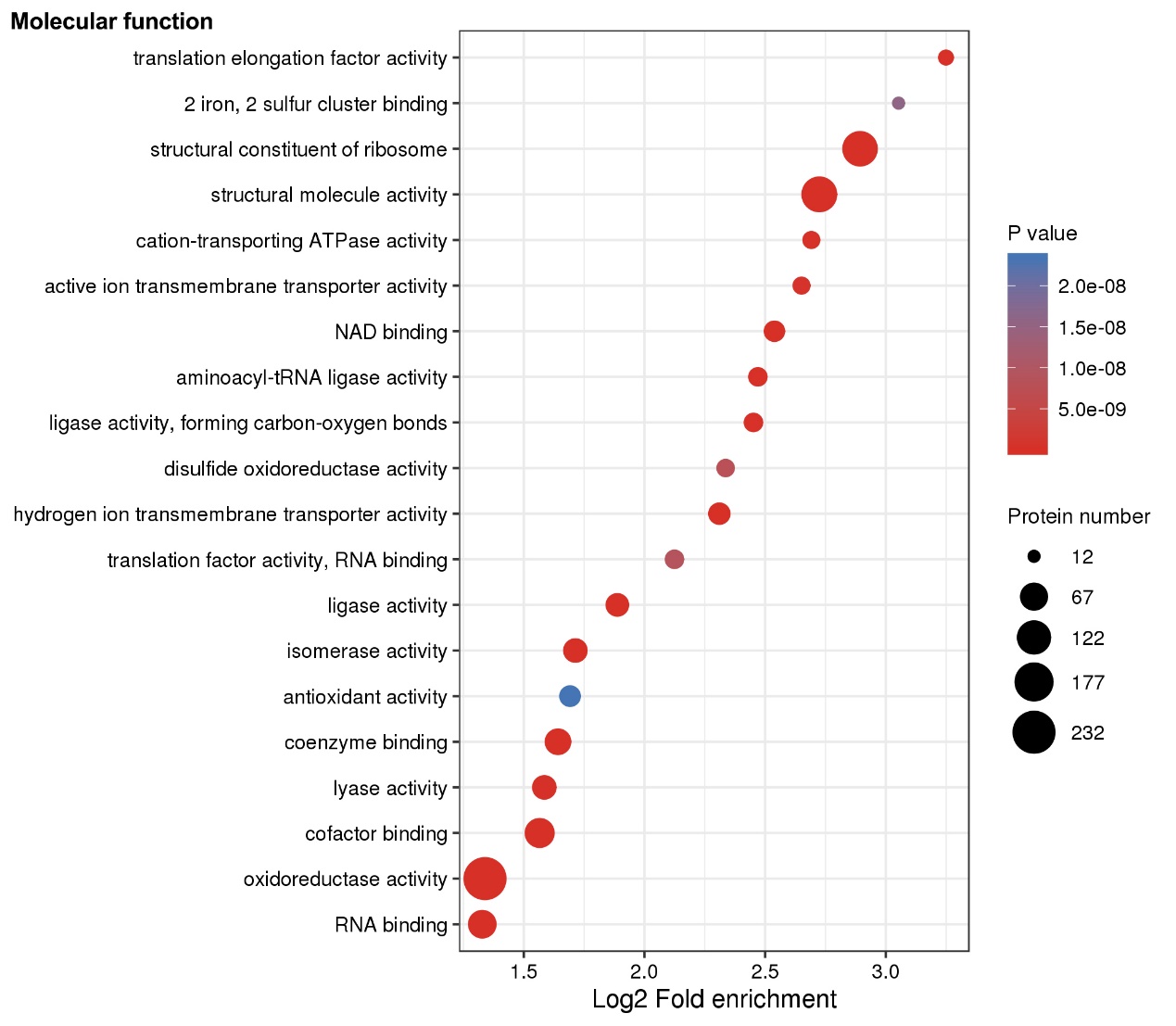


**Fig. S1.** The bubble chart shows the GO enrichment analyses of K_hib_-modified proteins in soybean leaves.


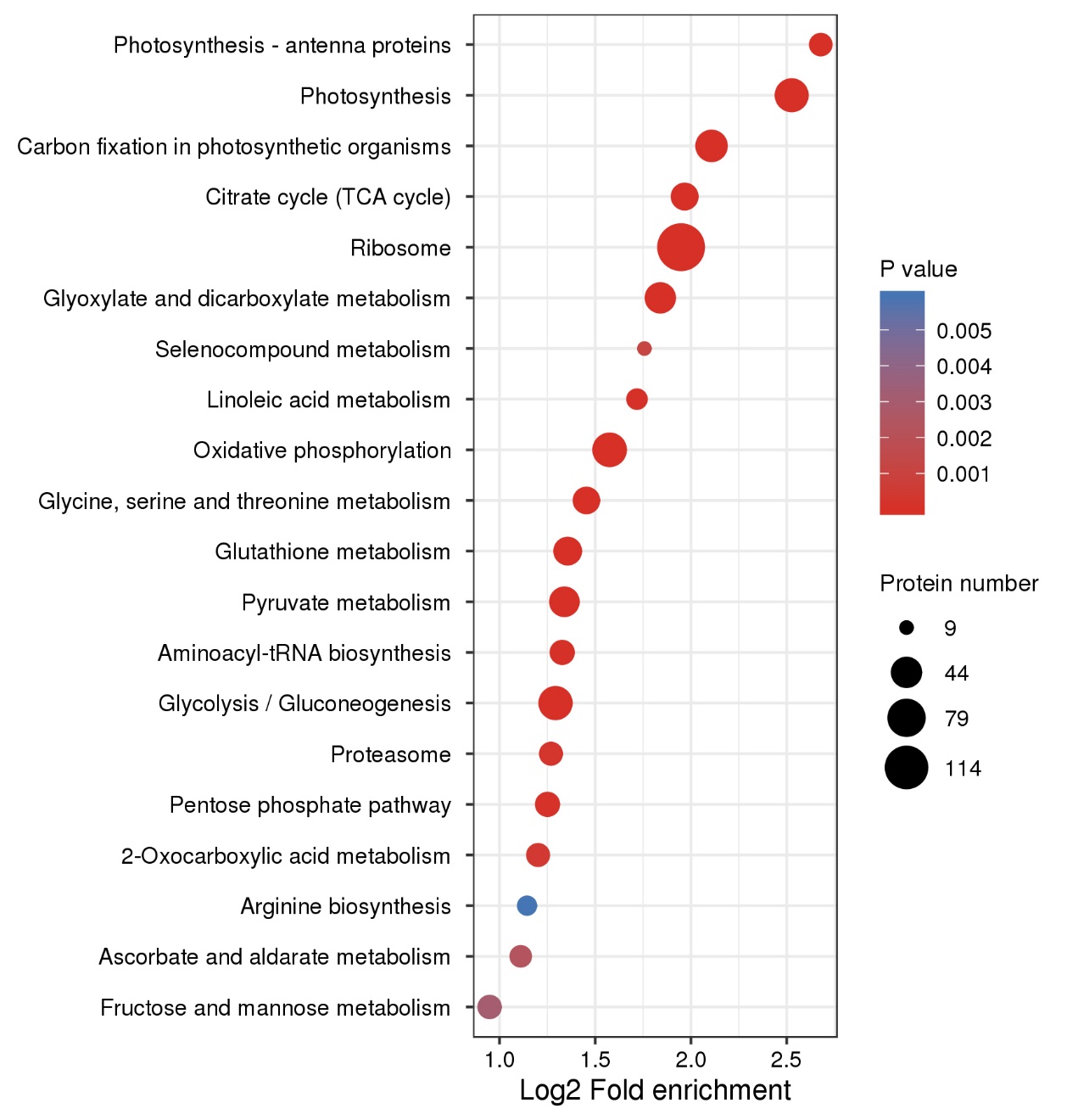


**Fig. S2.** The bubble chart shows the enrichment of KEGG pathway of K_hib_-modified proteins in soybean leaves.


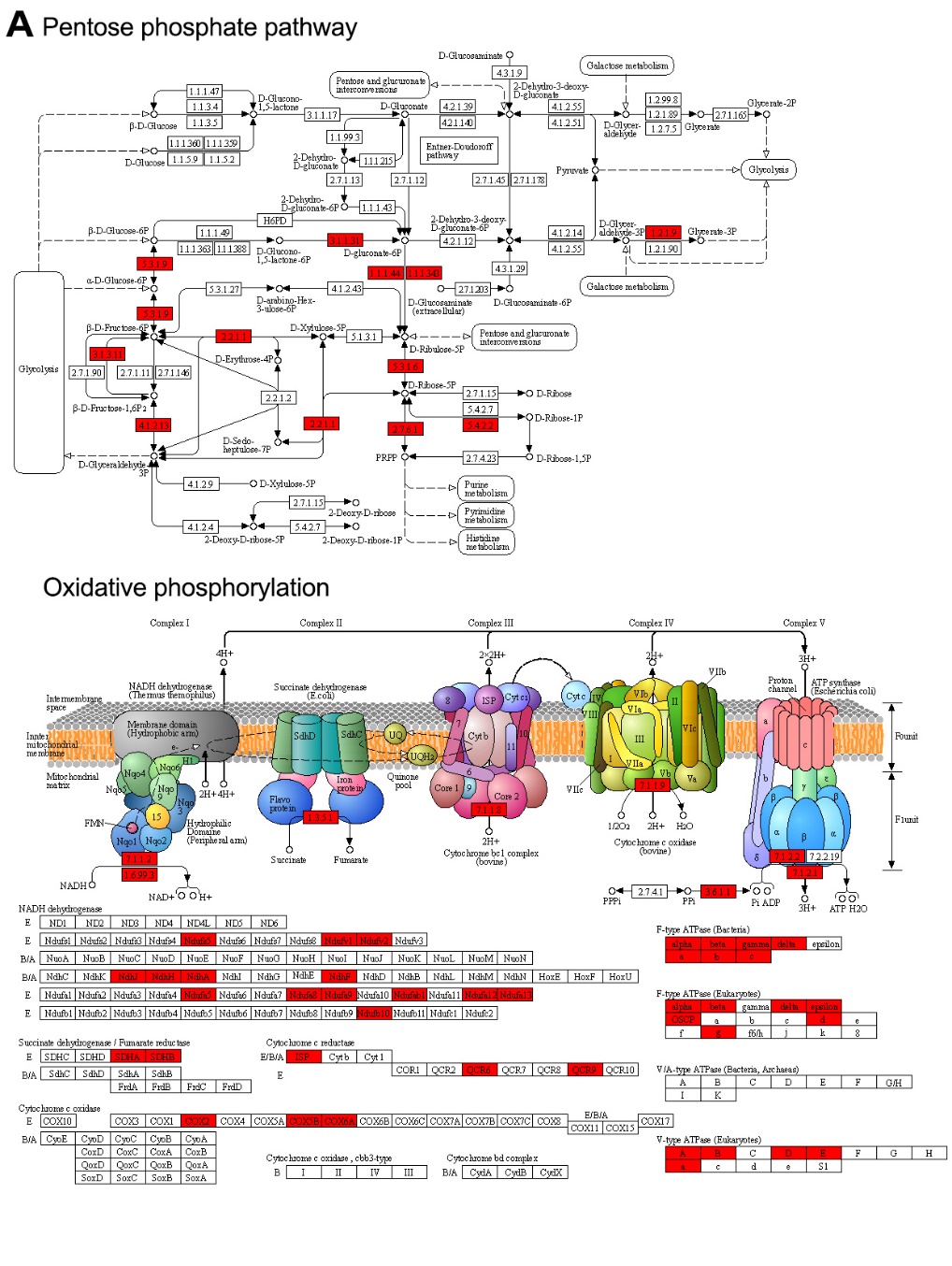

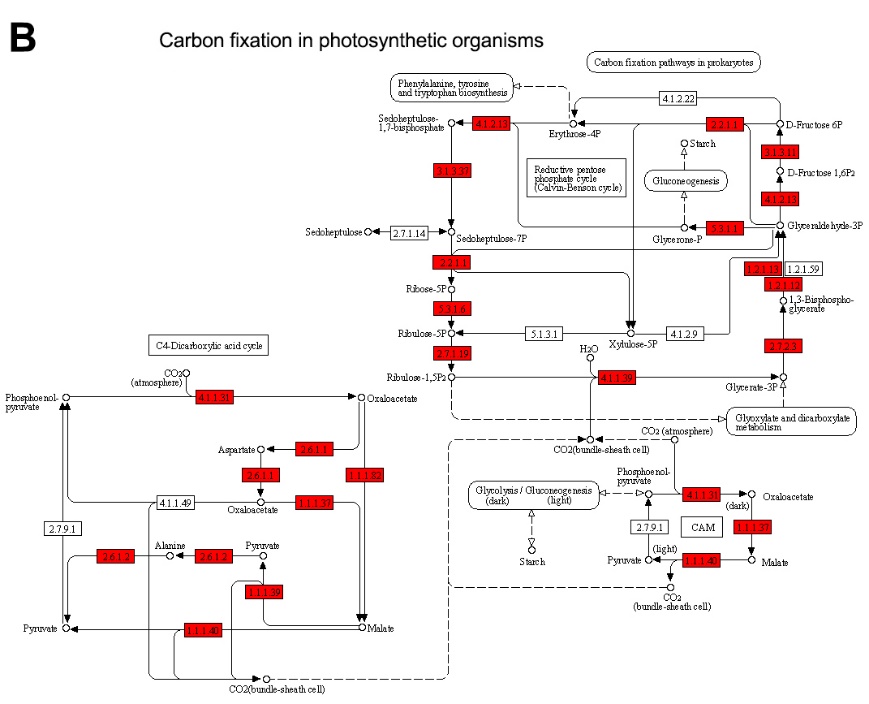


**
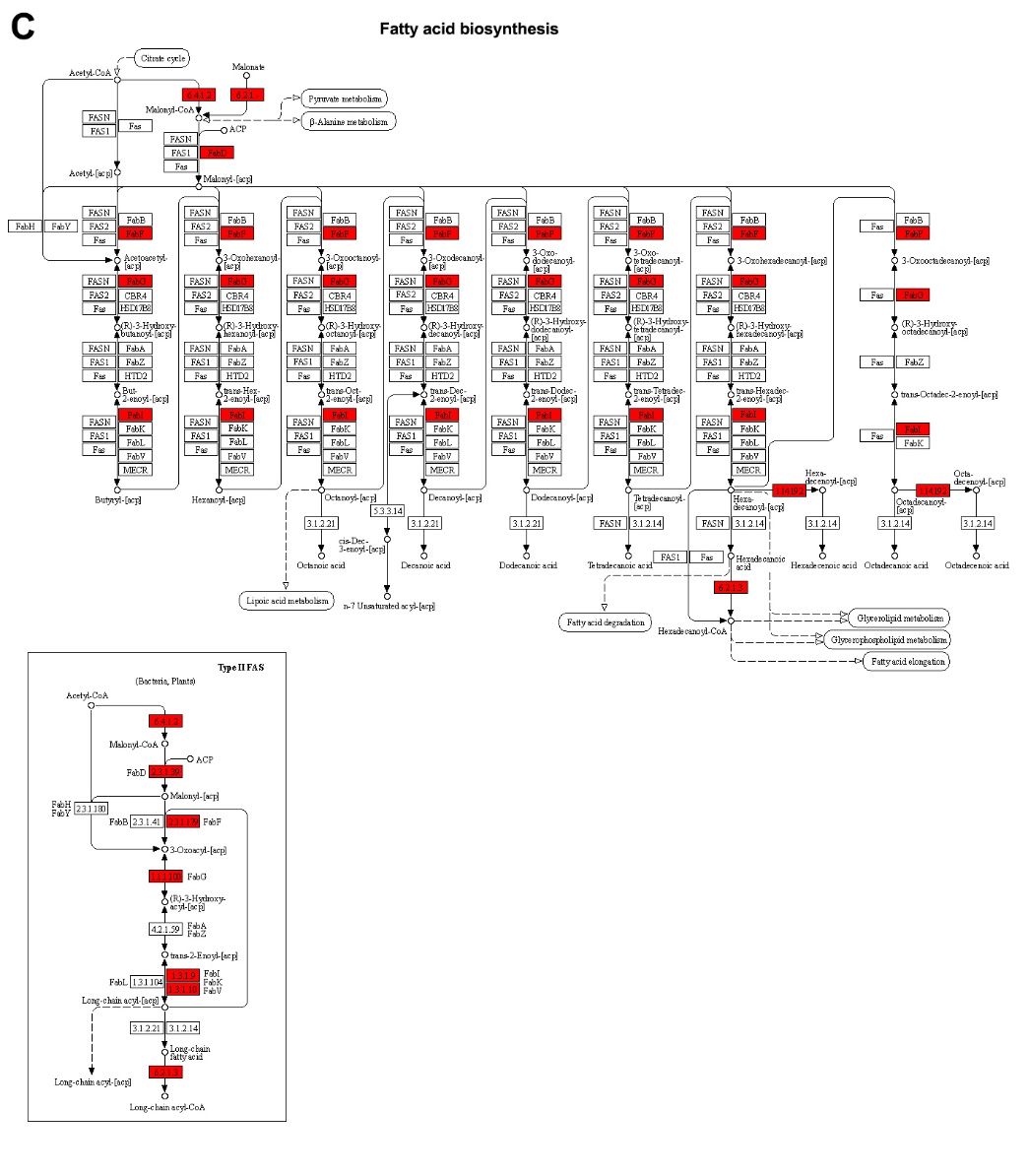
**

**Fig. S3.** Representative pathway maps of K_hib_-modified proteins. (A) K_hib_-modified proteins in central carbon metabolism, including pentose phosphate pathway and oxidative phosphorylation. (B) K_hib_-modified proteins in carbon fixation in photosynthetic organisms. (C) K_hib_-modified proteins in fatty acid biosynthesis. The identified K_hib_-modified proteins are indicated in boxes with a red background.

**
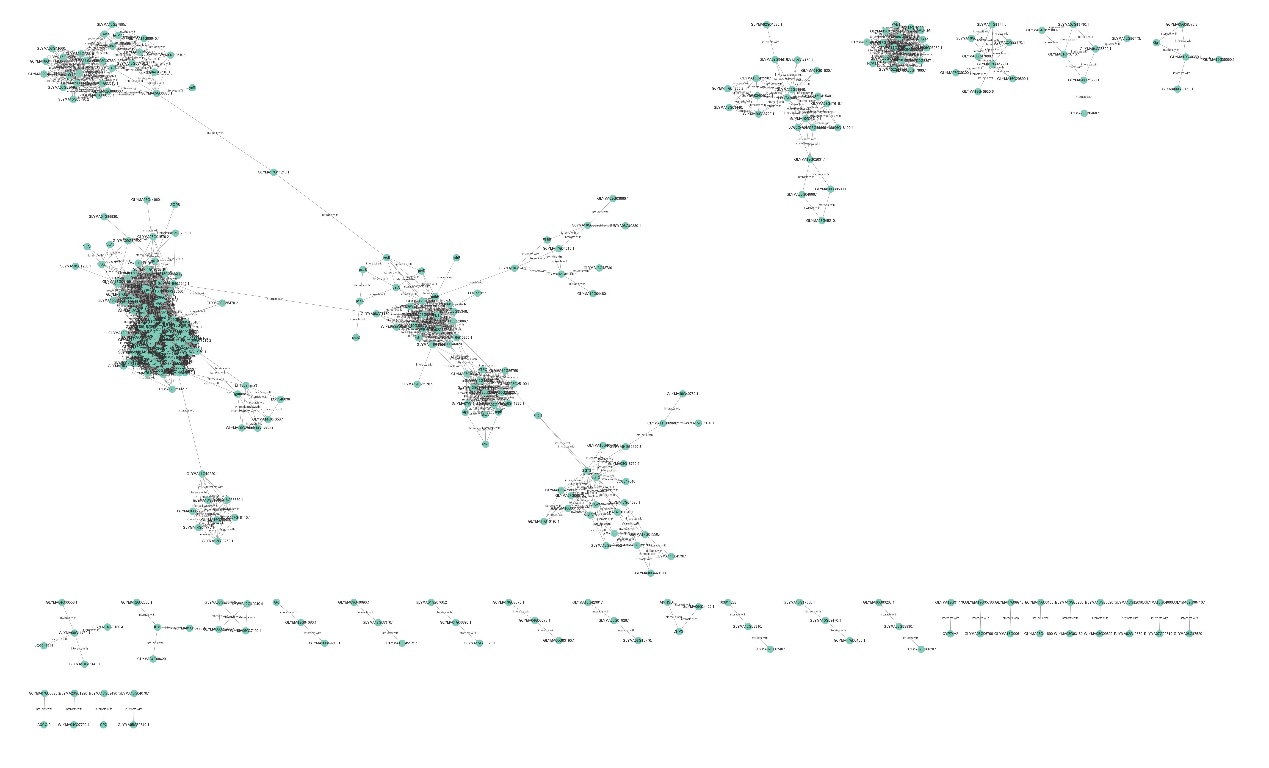
**

**Fig. S4.** The global PPI network of identified K_hib_-modified proteins in soybean leaves.

**2. Supplemental tables**

**Table S1**. All identified Khib sites and proteins that overlap in three replicates in soybean leaves.

**Table S2**. GO functional classification of K_hib_-modified proteins in soybean leaves.

**Table S3**. Subcellular localization distribution of K_hib_-modified proteins in soybean leaves.

**Table S4**. Secondary structure and surface accessibility distribution of K_hib_-modified peptides in soybean leaves.

**Table S5**. GO enrichment of K_hib_-modified proteins in soybean leaves.

**Table S6**. KEGG pathway enrichment of K_hib_-modified proteins in soybean leaves.

**Table S7.** Motif analysis of K_hib_-modified peptides in soybean leaves.

**Table S8**. The whole PPI network of K_hib_-modified proteins in soybean leaves.

(Note: all supplemental tables are showed in Excel files (.xlsx))
